# Supplementary material for: Selection and Evaluation of mRNA and miRNA Reference Genes for Expression Studies (qPCR) in Archived Formalin-Fixed and Paraffin-Embedded (FFPE) Colon Samples of DSS-Induced Colitis Mouse Model
Source: Biology (Basel). 2023 Jan 26;12(2):190. doi: 10.3390/biology12020190 (PMC9952917; doi:10.3390/biology12020190)
Supplement: Supplementary file 1 [file biology-12-00190-s001.zip › biology-2165068-supplementary.pdf]

Supplementary tables:

	Series/ experiment	F X (n)	M X(n)	DSS [%]	n days of DSS	n days/months of water	Histology
E1	Experiment March 2011 (Start 16. 3. 2011)	5 (10)		Control group	0	(EVT. 31. 3. 2011)	Normal
		1 (1)		2%	6	6 days (EVT. 29. 3. 2011)	Inflammation, erosion
		9 (9)		2%	6	7 days (EVT. 30. 3. 2011)	Inflammation, erosion
E3	Experiment May 2011 (Start 5. 5. 2011)	10 (10)		2%	5	22 days (EVT. 1. 6. 2011)	Mild inflammation
E4	Experiment December 2011 (Start 25. 11. 2011)	9 (9)		2,50%	5	7 days (EVT. 16. 12. 2011)	Severe inflammation
E2	Experiment Oct./Nov. 2013 (Start 3. 11. 2013)	5 (10)		Control group	0	(EVT. 28. 11. 2013)	Normal
		4 (5)		3%	5	10 days (EVT. 26. 11. 2013)	Inflammation, erosion
		5 (5)		3%	5	11 days (EVT. 27. 11. 2013)	Inflammation, erosion
E5	Experiment May 2012 (Start 9. 5. 2012)	9 (9)	6 (6)	2,50%	4	8 days (EVT. 21. 5. 2012)	Heterogeneous
E9	Experiment 2010 (Start 11. 11. 2010)		2 (2)	3%	5	0 days (EVT. 16. 11. 2010)	Heterogeneous
			2 (2)	3%	5	2 days (EVT. 18. 11. 2010)	Heterogeneous
			1 (2)	3%	5	3 days (EVT. 19. 11. 2010)	Heterogeneous
			1 (2)	3%	5	7 days (EVT. 23. 11. 2010)	Heterogeneous
			2 (6)	3%	5	29 days (EVT. 15. 12. 2010)	Heterogeneous
E7	Experiment Apr./Jun. 2012 (Start 26. 3. 2012)		7 (8)	Control group	0	(EVT. 20. 6. 2012)	Normal
			1 (1)	3%	5	5/6 days (EVT. 5/6. 4. 2012)	Severe inflammation
			8 (13)	3%	5	6 days (EVT. 6. 4. 2012)	Severe inflammation
			8 (12)	3%	5	3 months (EVT. 20. 6. 2012)	Moderate inflammation
E6	Experiment 2013 (Start 11. 11. 2013)		1 (3)	Control group	0	(EVT. 26. 11. 2013)	Normal
			2 (2)	Control group	0	(EVT. 13. 12. 2013)	Normal
			5 (5)	3%	5	10 days (EVT. 26. 11. 2013)	Moderate inflammation
			5 (5)	3%	5	24 days (EVT. 13. 12. 2013)	Moderate inflammation
			4 (4)	3%	5	3 months (EVT. 11. 2. 2014)	Heterogeneous
E8	Experiment 2014 (Start 7. 4. 2014)		5 (5)	3%	5	24 days (EVT. 8. 5. 2014)	Moderate inflammation

Supplementary Table S1: The data on archived FFPE colon samples of C57BL/6JOlaHsd mice involved in nine DSS experiments (not listed from 1 to 9) used in our study. Table shows number of FFPE samples (n) selected from each experiment (E1-E9), together with the information about DSS treatment (i.e. DSS and control groups, females and males and duration of inflammation (n days/months of water)). Legend: DSS, dextran sodium sulfate treatment; F, female; M, male, E1-9, experiment number; X, number of animals used in experiments; n, number of animals used in our study.

Gene / miRNA	Minimum		Maximum		Median		Mean		SD	
	C	DSS	C	DSS	C	DSS	C	DSS	C	DSS
EEF2	24.02	24.87	29.94	32.34	25.77	26.63	26.00	27.13	1.806	1.681
TBP^{+}	32.07	31.99	38.13	40.71	33.97	33.55	34.07	34.23	1.501	1.774
NONO	24.67	28.12	29.63	34.87	26.28	29.92	26.44	30.43	1.452	1.708
PPIA	25.49	24.43	34.58	33.13	28.20	26.47	28.44	27.07	2.625	2.201
RPLP0	23.76	23.95	31.88	32.92	26.45	26.59	26.64	26.89	2.403	2.420
miR-191-5p	27.63	27.31	30.20	32.29	28.17	28.54	28.45	28.59	0.727	0.873
miR-103a-3p	26.86	27.26	30.09	33.27	27.97	28.96	28.12	29.06	0.775	0.989
miR-16-5p	25.81	25.39	28.40	30.95	26.34	26.40	26.35	26.55	0.676	0.841
U6	26.04	24.79	28.62	32.43	26.76	27.16	26.93	27.23	0.732	1.315

Supplementary Table S2. Basic statistics of the Cq-values of candidate reference genes in archived FFPE colon samples of control (n=20) and DSS-treated (n=97) C57BL6/JOlaHsd mice. Legend: SD, standard deviation; *TBP*$^{+}$, 16 of 117 FFPE samples were undetermined for *TBP,* therefore, only 101 FFPE samples in which *TBP* was expressed were included in the calculation.

Gene	Minimum		Maximum		Median		Mean		SD	
	F	M	F	M	F	M	F	M	F	M
EEF2	24.02	25.70	27.56	32.34	25.72	27.98	25.76	28.05	0.800	1.672
TBP^{+}	31.99	32.99	35.36	40.71	33.01	35.16	33.19	35.51	0.767	1.742
NONO	24.67	26.06	32.16	34.87	28.90	30.65	28.70	30.75	1.736	2.228
PPIA	24.43	25.13	28.50	34.58	25.32	28.60	25.84	28.69	1.278	2.247
RPLP0	23.76	24.92	27.50	32.92	24.88	28.24	25.17	28.45	1.034	2.249
miR-191-5p	27.31	27.47	29.73	32.29	27.99	28.93	28.16	28.95	0.589	0.883
miR-103a-3p	27.26	26.86	31.67	33.27	28.34	29.25	28.50	29.28	0.836	1.033
miR-16-5p	25.39	25.66	27.61	30.95	26.00	26.82	26.09	26.98	0.462	0.838
U6	24.79	26.04	32.43	29.16	26.47	27.58	26.81	27.52	1.512	0.771

Supplementary Table S3. Basic statistics of the Cq-values of candidate reference genes in archived FFPE colon samples of control and DSS treated C57BL6/JOlaHsd mice for both sexes (females (n=57) and males (n=60)). Legend: F, female; M, male; SD, standard deviation; *TBP*$^{+}$*,* 16 of 117 FFPE samples were undetermined for *TBP,* therefore, only 101 FFPE samples in which *TBP* was expressed were included in the calculation.

**Intragroup variation calculated using NormFinder**				
Group	F Control group	M Control group	F DSS group	M DSS group
EEF2	0.086	0.028	0.143	0.172
TBP	0.173	0.351	0.089	0.843
NONO	0.015	0.202	0.040	0.163
PPIA	0.270	0.803	0.120	0.005
RPLP0	0.099	0.001	0.031	0.159
miR-191-5p	0.084	0.003	0.147	0.044
mir-103a-3p	0.002	0.137	0.058	0.089
miR-16-5p	0.005	0.020	0.257	0.057
U6	0.124	0.110	1.430	0.455
**Intergroup variation calculated using NormFinder**				
Group identifier	F Control group	M Control group	F DSS group	M DSS group
EEF2	-0.017	-0.278	0.316	-0.020
TBP	0.064	0.545	-0.484	-0.125
NONO	-1.181	-1.911	1.808	1.284
PPIA	0.952	1.212	-1.102	-1.062
RPLP0	0.183	0.432	-0.538	-0.077
miR-191-5p	0.170	0.038	-0.117	-0.091
mir-103a-3p	-0.261	-0.329	0.299	0.291
miR-16-5p	0.071	0.260	-0.207	-0.124
U6	0.020	0.031	0.025	-0.076

Supplementary Table S4. Intra- and intergroup variation of candidate reference genes in archived FFPE colon samples of control and DSS treated C57BL6/JOlaHsd mice of both sexes, calculated using NormFinder. Legend: F, female; M, male.

Supplementary Figures:

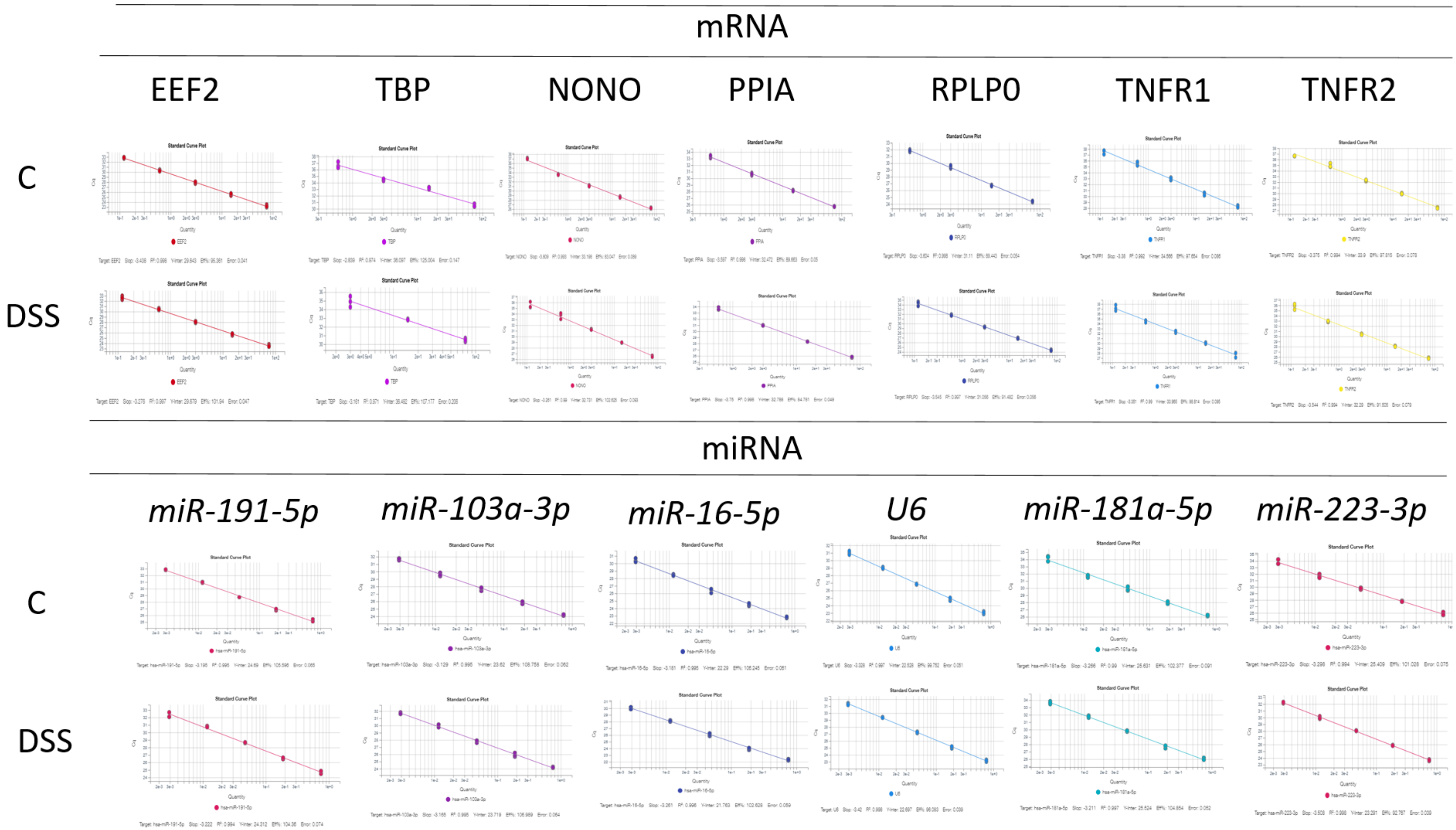

Supplementary Figure S1. Amplification efficiency (Eff = $[10^{(1/-S)-1}] \cdot 100\%$). Y-axis represents cycle threshold (Ct =Cq). X-axis represents log10 RNA dilution series. Calculations were made with Design & Analysis Software 2.5.1.

Influence of inter/intra-experimental factors on absolute and relative expression of TNFR2

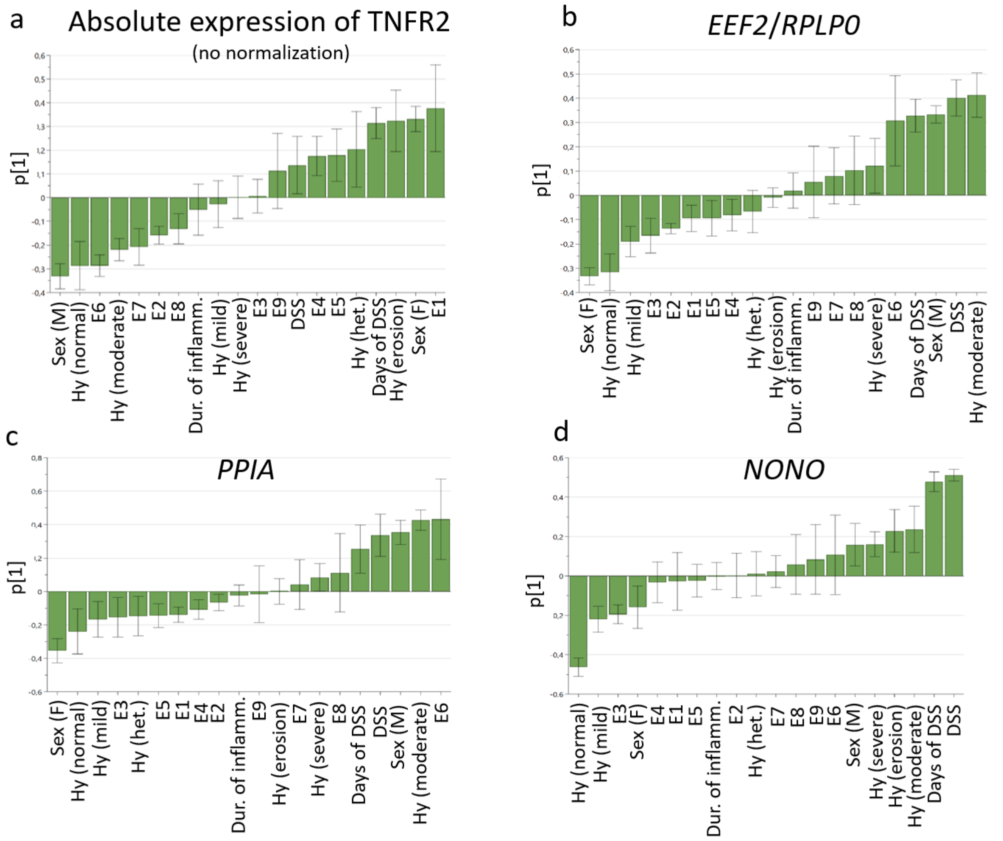

Supplementary Figure S2. Graphs represent influence of inter/intra-experimental factors on absolute (no normalization) and relative expression levels of *TNFR2* (normalized to the combination of reference genes *EEF2/RPLP0*, and *PPIA* and *NONO)*. Normalization of *TNFR2* reduced the influence of inter/intra-experimental factors on relative expression levels of *TNFR2*. Legend: F, female; M, male; E1-E9, experiment number; DSS, DSS treatment applied; duration of inflammation, days/months of water after DSS treatment; Hy, histology; normal, no lesion or inflammation; mild, moderate and severe refer to inflammation; erosion, destruction of epithelial layer of mucosa; heterogenous, includes both erosion and inflammation.

Influence of inter/intra-experimental factors on absolute and relative expression of miR-181a-5p

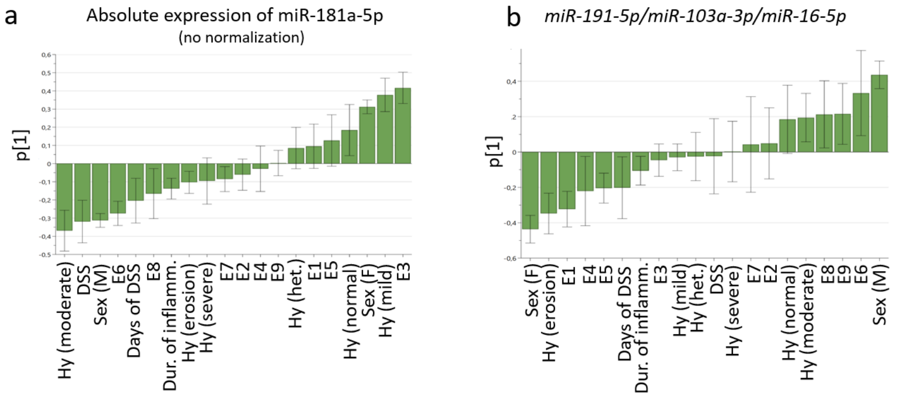

Supplementary Figure S3. Graphs represent influence of inter/intra-experimental factors on absolute (no normalization) and relative expression levels of *miR-181a-5p* normalized to the combination of reference genes *miR-191-5p/miR-103a-3p/miR-16-5p*.

Influence of inter/intra-experimental factors on absolute and relative expression of miR-223-3p

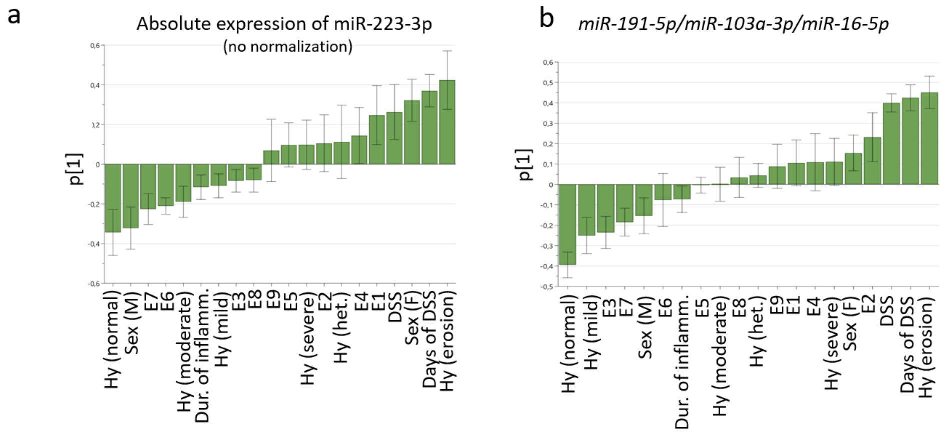

Supplementary Figure S4. Graphs represent influence of inter/intra-experimental factors on absolute (no normalization) and relative expression levels of *miR-223-3p* normalized to the combination of reference genes *miR-191-5p/miR-103a-3p/miR-16-5p*.

Supplementary Text:

Supplementary Text S1: PCA algorithm description

PCA uses covariance matrix as the method basis. Data is arranged in matrix where each column represents one variable (i.e. inter/intra-experimental factor) and each row represents one observation (i.e. sample). With categorical variables each category of a variable results in a separate column. Each row and column of a covariance matrix belongs to one variable, therefore, the main diagonal consists of variable variances non-diagonal elements represent variable covariances. Next, eigen vectors and eigen values are computed for the covariance matrix. Eigen values represent the variance of the data set that is explained by the corresponding eigen vector. In PCA, commonly eigen values are represented as shares of total data set variance each eigen vector component represents. Eigen vector actually represents coefficients for linear combination of original variables to form principal components, i.e. a set of uncorrelated new variables. For a data set of completely uncorrelated variables all eigen values would have the same value and each eigen vector would have one value 1 and the rest would be zeros, with one of different position for each eigen vector. Such a data set is only possible when experiments are properly designed, however, when experiments have already been performed, a randomized selection of samples is best available approach. For such data sets a number of significant principal components is smaller than the number of original variables. If the difference is not too big, such data set can be used to build relevant models.
